# Longitudinal course of affective disorders in patients presenting with catatonia in a psychiatric emergency setting

**DOI:** 10.1192/j.eurpsy.2021.335

**Published:** 2021-08-13

**Authors:** S. Varghese, S. Reddi, K.P. Muliyala, B. S, D. Guna

**Affiliations:** 1 Department Of Psychiatry, National Institute of Mental Health and Neuroscience, Bangalore, India; 2 Department Of Biostatistics, National Institute of Mental Health and Neuroscience, Bangalore, India

**Keywords:** Catatonia, Affective disorders, longitudinal course, emergency psychiatry

## Abstract

**Introduction:**

Catatonia, a complex psychomotor syndrome and psychiatric emergency, is encountered across various psychiatric disorders. Most findings have been derived in the context of schizohrenia, warranting more comprehensive understanding in affective disorders.

**Objectives:**

To evaluate the longitudinal course of affective disorders presenting with catatonia and factors influencing the same.

**Methods:**

Medical records of 439 patients presenting with catatonia to the psychiatry emergency from 2014 to 2017 were reviewed till June 2020. 135 patients with a final diagnosis of affective disorder (67 bipolar and 68 unipolar) were identified. Poisson regression and survival analysis were used for longitudinal data.

**Results:**

77.6% of bipolar patients were initially diagnosed under psychotic spectrum disorders compared to 3% in unipolar. Bipolar patients had a significantly younger age of first catatonic episode, earlier illness onset, and longer duration of illness. Survival analysis showed no significant difference between groups in time to recurrence of mood episode, readmission or catatonia relapse, with both groups demonstrating a greater likelihood of catatonia relapse in first 20 months. Poisson regression showed that bipolar patients had fewer catatonic relapses longitudinally over 2.5-6.5 years (RR: 0.64, CI: 0.43-0.96), but warranted more electroconvulsive therapy sessions for catatonic relapses (RR: 2.33, CI: 1.49-3.50), with fewer episodes resolving with lorazepam (RR: 0.62, CI: 0.40-0.95) compared to unipolar patients over same time period.

**Conclusions:**

Bipolar disorders appear to have an earlier onset but fewer episodes of catatonia over illness course. Poorer lorazepam response and higher number of ECT sessions for catatonia resolution longitudinally suggest a differential treatment response of catatonia in bipolar disorder.

**Disclosure:**

No significant relationships.

